# Unbiased Approach for Virus Detection in Skin Lesions

**DOI:** 10.1371/journal.pone.0065953

**Published:** 2013-06-28

**Authors:** Davit Bzhalava, Hanna Johansson, Johanna Ekström, Helena Faust, Birgitta Möller, Carina Eklund, Peter Nordin, Bo Stenquist, John Paoli, Bengt Persson, Ola Forslund, Joakim Dillner

**Affiliations:** 1 Departments of Laboratory Medicine, Medical Epidemiology & Biostatistics, Karolinska Institutet, Stockholm, Sweden; 2 Departments of Clinical Microbiology and Pathology, Karolinska Hospital, Stockholm, Sweden; 3 Department of Medical Microbiology, Skåne University Hospital, Lund University, Malmö, Sweden; 4 Dermatology Clinic, Läkarhuset, Gothenburg, Sweden; 5 Department of Dermatology and Venereology, Sahlgrenska University Hospital, Institute of Clinical Sciences at the Sahlgrenska Academy, University of Gothenburg, Sweden; 6 IFM Bioinformatics and Swedish e-Science Research Centre, Linköping University, Linköping, Sweden; National Institute of Health – National Cancer Institute, United States of America

## Abstract

To assess presence of virus DNA in skin lesions, swab samples from 82 squamous cell carcinomas of the skin (SCCs), 60 actinic keratoses (AKs), paraffin-embedded biopsies from 28 SCCs and 72 kerathoacanthomas (KAs) and fresh-frozen biopsies from 92 KAs, 85 SCCs and 92 AKs were analyzed by high throughput sequencing (HTS) using 454 or Ion Torrent technology. We found total of 4,284 viral reads, out of which 4,168 were Human Papillomavirus (HPV)-related, belonging to 15 known (HPV8, HPV12, HPV20, HPV36, HPV38, HPV45, HPV57, HPV59, HPV104, HPV105, HPV107, HPV109, HPV124, HPV138, HPV147), four previously described putative (HPV 915 F 06 007 FD1, FA73, FA101, SE42) and two putatively new HPV types (SE46, SE47). SE42 was cloned, sequenced, designated as HPV155 and found to have 76% similarity to the most closely related known HPV type. In conclusion, an unbiased approach for viral DNA detection in skin tumors has found that, although some new putative HPVs were found, known HPV types constituted most of the viral DNA.

## Introduction

Human papillomaviruses (HPVs) are a large and diverse group of viruses with more than 150 completely characterized types, with new HPV types being continuously found [1]–[12]. HPVs infect keratinocytes in mucosa or skin. There are five major HPV genera: Alphapapillomavirus, Betapapillomavirus, Gammapapillomavirus, Mu papillomavirus and Nu papillomavirus. HPV types belonging to different genera have less than 60% similarity, based on the nucleotide sequence of the capsid protein L1. Different viral species within a genus share between 60 and 70% similarity. A novel HPV type has less than 90% similarity to any other HPV type [13]. Novel HPV types are given a number only after the whole genome has been cloned and deposited with the International HPV Reference Center [6], [13].

HPVs cause a wide range of diseases from benign lesions to invasive tumours [14], [15]. The oncogenic mucosal HPV types in the Alphapapillomavirus genus are a major cause of cervical cancer [16], as well as anal, vulvar, and oral cancers [17]. There are also benign mucosal HPV types in the Alphapapillomavirus genus that cause benign genital condylomas [6].

The cutaneous HPV types are commonly found in several skin lesions such as benign skin warts [18], actinic keratoses (AKs), non-melanoma skin cancers (NMSCs) [17] and keratoacanthomas (KAs) [19], [20]. Cutaneous HPV types are also commonly detected on healthy skin [21].

NMSCs such as squamous cell carcinoma (SCC) and basal cell carcinoma (BCC) are two of the most prevalent cancers among Caucasian populations worldwide [22]. Ultraviolet radiation is a well-known risk factor [23], but there may also be other risk factors [24], [25]. The increased incidence of SCC in immunocompromised individuals has suggested that an infection may be involved [24], [26]–[28], with HPV being the most commonly studied candidate infectious agent [20], [29].

Skin samples from SCC, AK, seborrhoeic keratosis (SK), and BCC commonly contain multiple HPV types, but typically at very low viral loads [4], [5], [7], [19], [30], [31]. Conventional methods for virus detection suffer from low throughput and are furthermore biased towards identifying sequences homologous to the PCR primers used. There are several examples of NMSC lesions that contain very high amounts of previously uncharacterized HPV types that may escape detection by PCR systems based on known HPV sequences [7], [30]. This has made it difficult for epidemiologic studies to perform an unbiased assessment of which HPV types are present in healthy skin as compared to benign tumors or NMSCs.

High throughput sequencing (HTS) can be used for an increased sensitivity in HPV detection by sequencing of general HPV-PCR amplimers [11]. However, HTS can also be used to obtain a comprehensive and more unbiased sequencing of the DNA present in a sample, without any prior PCR or other prior information about sequences [32]. The HTS technology has been increasingly used for discovery of new viruses [12], [33]–[36]. The aim of this study was to apply the HTS technology for an unbiased analysis of which known and unknown HPV types are common in SCCs, AKs, and KAs.

## Materials and Methods

### Patients

We used four different patient series. One hundred and thirty-one patients registered with non-melanoma, non-basal cell skin cancer (ICD7°code 191) in the Swedish Cancer Registry with formalin-fixed paraffin-embedded (FFPE) biopsies stored at the Pathology Department of Malmö University Hospital, Sweden, were identified and re-reviewed by an expert pathologist [37]. We included 28 specimens that were classified as SCC and 72 specimens re-classified as KA. The paraffin blocks were sectioned and DNA extracted as described [37].

At the Department of Dermatology and Plastic Surgery at the Norwegian National Hospital, Oslo, Norway, biopsies from 92 KAs from both immunosuppressed and immunocompetent patients were collected. The DNA was extracted using the QIAampDNA Minikit (Qiagen, Hilden) [19].

From a hospital-based case-control of NMSCs, premalignant and benign lesions [29], conducted in Sweden and Austria, we included samples collected from immunocompetent patients with SCCs (n = 85), AKs (n = 92), BCCs (n = 118) and SKs (n = 46). All patients provided four different samples: swab samples from the top of the lesion and from healthy adjacent skin, as well as a biopsy of the lesion and from healthy adjacent skin. The swab samples were collected by a pre-wetted (0.9% NaCl) cotton-tipped swab that was rolled on the lesion (within margins of the lesion) and suspended in 1 ml of saline.

From a Swedish hospital-based study, we included 69 immunocompetent patients (SCC (n = 35), AK (n = 22), BCC (n = 3), KA (n = 8) and SK (n = 1)). All patients donated biopsies and swab samples in the same manner as described above. For the latter 2 series the biopsies were taken from the skin after tape-stripping, to remove possible environmental contaminations [21].

### Ethics statements

Written informed consent was obtained from participants. The study adhered to the declaration of Helsinki and was approved by the Ethical Review Committees of Karolinska Institute and of Lund University (Sweden), Medical University Vienna (Austria) and Institutional Review Board in Oslo (Norway).

### Sample preparation

Three different sample preparation methods were used: E-gel followed by whole genome amplification (WGA), ultracentrifugation followed by WGA and direct WGA of the sample.

#### Separation of long chromosomal DNA from shorter DNA using E-gel

Two to four µl of each sample were mixed according to diagnosis and sample type to pools of 6 to 11 patients each. The pools were run on an E-gel (Invitrogen, Carlsbad, CA) where DNA was separated by gel electrophoresis. DNA in the size range from ∼3 to 10 kb was eluted from the gel.

To obtain sufficient amounts of DNA for the 454 sequencing, we performed whole genome amplification (WGA) using GenomiPhi High Yield (GE Health Care, Buckinghamshire, United Kingdom) on all pools described above. The manufactureŕs protocol was followed, with some modifications. Five µl of sample was mixed with 1 µl 10× BSA (New England Biolabs) and 22.5 µl of sample buffer. The mix was incubated at 95°C for 3 min and then cooled on ice. For the amplification reaction 22.5 µl reaction buffer was mixed with 2.5 µl enzyme mix and added to the samples, incubated at 30°C for seven hours and inactivated at 65°C for 10 minutes. Sequencing products were dissolved by diluting the samples 1∶2 in TE-buffer (10 mM Tris-HCl, 0.1 mM EDTA, pH 8.0) and rotated on an orbital shaker (500 rpm) over night. Prior to sequencing, the smaller pools of 6 to 11 patients were mixed into seven pools (28 paraffin embedded samples from SCCs, 72 paraffin embedded samples from KAs, 92 fresh frozen KA biopsies from Norway, swab samples from the top of the lesion from 82 SCCs and 60 AKs, 41 fresh frozen biopsies from SCCs negative for HPV in a previous study [29], 44 fresh frozen biopsies from SCCs positive for HPV in a previous study [29] and 92 fresh frozen biopsies from AKs).

#### Separation of viral capsids from human chromosomes using ultracentrifugation

Swab samples from the top of the lesion from 82 SCC patients and 60 AK patients were prepared for density gradient ultracentrifugation by mixing 2 µl of each sample into 4 pools of about 35 patients each. Optiprep density gradient medium (Sigma) was used in concentrations of 39%, 33%, and 27%, as described [38]. The gradient layers had a volume of 300 μl. After adding the pools to the top of the gradient, the 5 ml polyallomer ultracentrifuge tube was filled with standard PBS. Centrifugation was performed with a Sorvall Discovery M120 SE centrifuge with swing-out rotor S52-ST at 50 000 g for 3:30 h. After the centrifugation, the bottom of the polyallomer tube was punctured with a needle and fractions collected. The first 100 μl fraction was discarded (where the human DNA is sedimented [39]) and the next fraction of 350 μl was collected (where virus particles but no human DNA is sedimented [39]). DNA was extracted with MagnNA Pure LC using the Total Nucleic Acid kit (Roche), Before sequencing, amplification using GenomiPhi High Yield was performed using five µl of the extracted DNA from each of the four pools.

#### Pooling of samples

DNA was amplified using GenomiPhi High Yield on each small pool of 8 to 23 patients before samples were pooled into seven larger groups (28 paraffin embedded samples from SCCs, 72 paraffin embedded samples from KAs, 92 fresh frozen KA biopsies from Norway, swab samples from the top of the lesion from 82 SCCs and 60 AKs, 41 fresh frozen biopsies from SCCs negative for HPV in a previous study [29], 44 fresh frozen biopsies from SCCs positive for HPV in a previous study [29], and 92 fresh frozen biopsies from AKs) and sent for 454 sequencing. The pool of swab samples of SCCs & AKs was also sequenced using Ion Torrent 300 and 400 bp sequencing kits.

### Sequence analysis

Sequences were obtained from the 454 Genome Sequencer FLX Titanium ( Roche) at the Science for Life Core Facility in Stockholm, Sweden and Ion Torrent PGM sequencer (Life Technologies) at the Science for Life Core Facility in Uppsala, Sweden. Multiplex identifiers (MIDs), included in the 454 adaptors, were used to assign the sequences obtained to the originating sample.

Duplicated sequences were identified using cdhit-454 algorithm [40] and were removed from further analysis. The remaining sequences were quality checked and base pairs with Phred quality score of less than 15 were defined as ambiguous. A Phred quality score of 15 equals to a base calling accuracy of 97% or 3 errors in 100 bases [41]). Tails containing ambiguous base pairs were trimmed off. Thereafter, sequences with the length of ≥80 base pairs and containing unambiguous base pairs of at least 80% of their length were selected for further analysis.

The selected high quality reads were compared to viral, human and bacterial DNA in GenBank, as well as to sequencing reads from negative control samples (water) [39] using the SSAHA2 software [42]. Reads with at least 90% identity over 80% of their length to human or bacterial DNA or to sequencing reads from negative control samples (water) were removed from further analysis. Remaining sequences were processed for assembly of contiguous sequences (contigs). Genome assembly was performed *de novo* using the sequence assembly software MIRA (minimum overlap of 40 base pairs with at least 98% identity) [43] and Celera Assembler's CABOG pipeline (minimum overlap of 40 base pairs with at least 98% identity) [44]. CABOG generated longer and fewer contigs than MIRA and all final analyses were conducted using CABOG. After manual inspection and finishing of assembly using the visual analytics tool Hawkeye [45], all contigs and unassembled sequences (singletons) were compared against GenBank nucleotide database using the BLASTn algorithm [46] (reward for nucleotide match = 1; penalty of nucleotide mismatch = 1; cost to open a gap = 0; cost to extend a gap = 2; e-value≤e^−4^) to classify them as papillomaviruses-related or not. To identify possible artifactual “chimeric” sequences, that contain sequences originating from different viruses, all papillomaviruses-related contigs and singletons were checked as described [36]. Shortly, the sequence that aligned to its most closely related sequence in GenBank was divided into three equal segments. If at least one of the segments differed in similarity to the corresponding overlapping parts with more than 5% (for example if segment 1 was 88% similar and segment 2 was 94% similar) the sequence was considered as a “possible chimera”.

All analyses were performed using in-house R (www.R-project.org) and python (www.python.org) scripts. Coverage plots were generated using Circos visualization tool [47].

### Screening of HPV155 and SE46

Prevalence of HPV155 (SE42) and SE46 was investigated using real-time PCR in the pool of swab samples of SCCs & AKs and the original samples from the hospital-based case-control studies where sufficient amounts were available. For HPV155, 1364 samples from 341 patients with biopsies and swab samples from lesions and healthy skin (SCC, n = 89; AK, n = 77; SK, n = 48; BCC, n = 119; and KA, n = 8) were analysed. For SE46, we tested 1368 samples from 341 patients with biopsies and swab samples from lesions and healthy skin (SCC, n = 89; AK, n = 76; SK, n = 47; BCC, n = 121; and KA, n = 8). Insufficient amount of samples for some patients resulted in that the number are not exactly the same.

Primers and probes, were designed using the Primer Express Software for Real-Time PCR, v.3.0 (Applied Biosystems); HPV155-F: 5′-ATGGAAGGTTAGAGGGTTAGAAGTCATT-3′, HPV155-R: 5′-TTTGTCTGTTATCAGTACTAGGTTGGAAA-3′, HPV155 probe: 5′-FAM-AATGACACTGAAAATCCAAA-NFQ-3′; SE46-F: 5′-CACTTCTTACTCCGGGAAGACCT-3′, SE46-R: 5′-CGACCTCCGGTTTTTGGAGT-3′, SE46 probe: 5′-FAM-CAAAGTCCCCGTATCCA-NFQ-3′, (the MGB probes were produced by Applied Biosystems, UK, and the primers by Eurofins MWG Operon, Germany). Standard curves used serial dilutions from 500,000 to 0.5 copies/PCR of purified viral DNA from plasmids containing the real-time PCR-fragment of HPV155 or SE46 in a background of 10 ng/µl human placental DNA (Type XIII, Sigma-Aldrich). The PCR mixtures were prepared in a clean room, and contained in a total of 25 µl; 2.5 µl sample, 0.4 µM of each primer, 0.04 µM MGB-probe, 1x TaqMan® Universal PCR Master Mix (Applied Biosystems) in sterile water (Sigma-Aldrich, Steinheim, Germany). Samples were added to the PCR mixture in a separate room. Water and TE-buffer (10 mM Tris-HCl, 0.1 mM EDTA, pH 8.0) were used as non-template control in each run. The PCR-analyses were carried out in a 7500 Fast Real-Time PCR System, using the 7500 Software v.2.0.5 (Applied Biosystems), with the following temperature settings; 2 min at 50°C and 10 min at 95°C, followed by 50 cycles at 95°C for 15 s and 60°C for 1 min. The threshold was set to 0.1 ΔRn (Rn is the fluorescence of the reporter dye divided by the fluorescence of the passive reference. For ΔRn the baseline fluorescence has been subtracted). Samples were run in triplicate and scored positive if at least two tests became positive.

The mean viral copy number of HPV155 and SE46 were calculated, and was for the biopsies normalised against the amount of human cells as determined in a single test by the use of a real-time PCR of the human β-globin gene [48]. The β-globin assay used a standard curve with serial dilutions from 100,000 to 1 copy per PCR of human placental DNA (Type XIII, Sigma-Aldrich) diluted in sterile water (Sigma-Aldrich).

### Characterization of HPV155

The genome of HPV155 was amplified and cloned from a top-of-the-lesion swab from a patient with AK, in three overlapping fragments. The fragments were amplified using the Expand High Fidelity PCR System (Roche), where individual PCR-programs were set for each fragment according to manufacturer's instruction. Fragment 1 (bp 4973–1635) was amplified using the primers HPV155 1-F 5′- GTGTCTTCGCATGTGGTTTTAACTT-3′ and HPV155 1-R 5′- TGGTCATATGCAAACTGTACCATTTT-3′. The PCR-program was set as follows; 94°C for 2 min, followed by 40 cycles at 94°C for 15 s, 50°C for 30 s, 68°C for 3 min +5 s/cycle, and 68°C for 7 min. The second fragment (bp 1298–3659) was amplified using HPV155 2-F 5′-ACAGGATGAACTATGTGAAAGCTCAA-3′ and HPV155 2-R 5′-GAATCCAACTGACCCAATGTGTACT-3′, whilst fragment 3 (bp 3273-5263) was amplified using HPV155 3-F 5′-ACGACGACAGCGACGAGAA-3′ and HPV155 3-R 5′-TTTCCACTGTTCGGTAGCCAA-3′. Fragments 2 and 3 were both amplified using 94°C for 2 min, 40 cycles at 94°C for 15 s, 55°C for 30 s, 72°C for 2 min +5 s/cycle, and 72°C for 7 min.

The 25 µl PCR mixtures contained 2.5 µl sample, 0.3 µM of each primer, 3.5 mM Mg^+2^, 0.3 mM of each dNTP, 1x Expand High Fidelity Buffer without MgCl_2_ (Roche), and 0.62 U Expand High Fidelity Enzyme mix (Roche) in water (Sigma-Aldrich, Steinheim, Germany).

The fragments were cloned using the TOPO TA Cloning kit (Invitrogen) using the pCR®2.1-TOPO® vector, according to manufacturer's instructions. The fragments were sequenced using both conventional primer walking and using the 454 FLX sequencer GS Junior (Roche). The 454 sequencing of the HPV155 plasmids was performed in-house, using the manufactureŕs instructions. The primers for the Sanger sequencing were from Eurofins MGW Operon (Germany).

## Results

### Sequence analysis

The sequencing using 454 technology of swab samples from 82 SCCs and 60 AKs, paraffin-embedded biopsies from 28 SCCs and 72 KAs and fresh-frozen biopsies from 92 KAs, 85 SCCs and 92 AKs obtained 1,359,108 sequence reads. Ion Torrent PGM sequencing of swab samples from 82 SCCs and 60 AKs, using 300 and 400 bp sequencing kits obtained 912218 and 381017 sequencing reads, respectively (Table 1). After removal of duplicated sequences and filtering based on quality scores and read length, 1,116,074, 717880 and 274090 reads remained for 454, Ion Torrent 300 bp kit and Ion Torrent 400 bp kit respectively. They were classified into “human”, “bacterial”, “viral”, “negative control (water)” [39], “other” and “unknown” (Table 1). As “other”, we have classified reads with similarities to other sequences present in GenBank (e.g. plants, plant viruses, synthetic constructs). Sequences, which did not give any hits to sequences deposited in GenBank, were classified as “unknown” (Table 1). Previously, we reported presence of bacterial related sequences and sequences classified as “other” and “unknown” in negative control samples (water) after 454 sequencing [39]. Such background sequences might be present due to background reactivity of Phi29 polymerase reaction [49] or environmental contamination. As water controls were found to be uniformly negative for viral sequences [39], we removed all sequence reads that were related to the sequences found in water control.

**Table 1:** Number of sequence reads classified by taxonomies.

Disease^1^	SCC		KA		KA		SCC & AK					SCC		SCC		AK	
Sample type	Paraffin		Paraffin		Fresh Frozen		Swab samples from the top of lesions					Fresh frozen SCCs previously PCR-negative for HPV [29]		Fresh frozen SCCs previously PCR-positive for HPV [29]		Fresh Frozen	
Pre-amplification treatment	E-gel+ WGA	WGA	E-gel + WGA	WGA	E-gel + WGA	WGA	E-gel + WGA	UC + WGA	WGA			E-gel + WGA	WGA	E-gel + WGA	WGA	E-gel + WGA	WGA
Number of patients^2^	28	28	72	72	92	92	82 SCCs and 60 AKs	82 SCCs and 60 AKs	82 SCCs and 60 Aks			41	40	44	41	92	92
Sequencing platform	GSFLX	GSFLX	GSFLX	GSFLX	GSFLX	GSFLX	GSFLX	GSFLX	GSFLX	Ion PGM 300bp kit	Ion PGM 400bp kit	GSFLX	GSFLX	GSFLX	GSFLX	GSFLX	GSFLX
Total number of row reads	22284	67916	6203	54205	149535	172123	67636	69072	121752	912218	381017	79080	157956	44457	109038	98969	138882
Number of reads after de-duplication, quality and length filtering	17532	59699	4916	45484	112324	151009	55393	49491	110837	717880	274090	45245	138263	34859	92004	77205	121813
Human	10578	26106	3765	13179	112146	150818	23570	1052	76598	554860	208995	28821	138068	34796	91764	76854	121661
Bacteria	2600	8869	683	13503	56	66	20388	30244	26772	131317	50289	11352	69	27	184	163	30
Virus	1	1	0	252	5	4	34	51	380	2750	765	4	0	0	1	29	7
Negative (water) control [39]	1	2916	0	2320	0	1	3	36	4	6	0	0	1	0	1	0	0
Other	2303	3056	321	2433	81	60	9462	15094	2456	9636	2723	1765	59	29	25	128	70
Unknown	2049	18751	147	13797	36	60	1936	3014	4627	19311	11318	3303	66	7	29	31	45

1 SCC = squamous cell carcinoma, AK = actinic keratosis, KA = keratoachantoma, WGA =  whole genome amplifications.

2 Some samples were available in low volume and could not be included in all experiments.

Of the 769 viral reads, identified by 454 sequencing, 86% (662) reads were classified as related to HPV (Table 2). Most HPV-related sequences were found in the pool of formalin-fixed paraffin embedded KAs and in the pool of top-of-the-lesion swab samples from SCCs and AKs (250 and 378 reads, respectively). Ion Torrent PGM sequencing of the pool of top-of-the-lesion swab samples from SCCs and AKs identified a total of 3515 (300 bp kit –2750; 400 bp kit – 765) viral reads, where 99,78% (2744) and 99,6% (762) where HPV related in 300 bp and 400 bp sequencing runs, respectively (Table 2). We identified contigs and singletons with lengths ranging from 83 bp up to 7359 bp. Non-HPV-related viruses included human herpesvirus 8, Epstein-Barr virus, human endogenous retrovirus, Merkel cell polyomavirus, human polyomavirus 6 and Torque Teno virus (Table 2). We detected sequences from 15 known HPV types (HPV8, HPV12, HPV20, HPV36, HPV38, HPV45, HPV57, HPV59, HPV104, HPV105, HPV107, HPV109, HPV124, HPV138, HPV147) and four previously described putative HPV types (HPV 915 F06°007 FD1, FA73, FA101 and SE42) (Table 2). For FA101, we obtained a 7359 bp long contig, representing a complete genome, which was formed by 247 reads from the pool of paraffin embedded KAs (Table 2). HPV38, HPV45, HPV59, HPV105, HPV107, HPV138, HPV147, and FA73 were only found as one singleton sequence each. SE42 is a recently described subgenomic sequence, originally detected using pre-amplification by a degenerate HPV primer pair followed by HTS [11]. One hundred fifty-six reads from 454 sequencing were assembled into two contigs, related to SE42 genome, with the length of 763 bp (from E1 ORF part) and 6552 bp (from E2, E4, L2, L, E6, E7, L1 ORF part). Ion Torrent PGM sequencing (both 300 bp and 400 bp kit) detected complete genome of SE42.

**Table 2:** Number of reads related to viruses in each sample group.

Disease^1^	SCC		KA		KA		SCC & AK					SCC		SCC		AK	
Sample type	Paraffin		Paraffin		Fresh Frozen		Swab samples from the top of lesions					Fresh frozen SCCs previously PCR-negative for HPV [29]		Fresh frozen SCCs previously PCR-positive for HPV [29]		Fresh Frozen	
Pre-amplification treatment	E-gel +WGA	WGA	E-gel +WGA	WGA	E-gel +WGA	WGA	E-gel + WGA	UC + WGA	WGA			E-gel + WGA	WGA	E-gel +WGA	WGA	E-gel +WGA	WGA
Number of patients^2^	28	28	72	72	92	92	82 SCCs and 60 AKs	82 SCCs and 60 AKs	82 SCCs and 60 AKs			41	40	44	41	92	92
Sequencing platform	GSFLX	GSFLX	GSFLX	GSFLX	GSFLX	GSFLX	GSFLX	GSFLX	GSFLX	Ion PGM 300bp kit	Ion PGM 400bp kit	GSFLX	GSFLX	GSFLX	GSFLX	GSFLX	GSFLX
HPV8	0	0	0	0	0	0	0	0	189	1360	429	0	0	0	0	0	0
HPV12	0	0	0	0	0	0	0	0	1	8	4	0	0	0	0	0	0
HPV20	0	0	0	0	0	0	0	0	2	1	0	0	0	0	0	0	0
HPV36	0	0	0	0	0	0	0	0	0	0	0	0	0	0	0	24	2
HPV38	0	0	0	0	0	0	0	0	0	1	0	0	0	0	0	0	0
HPV45	0	0	0	0	0	0	0	0	0	1	0	0	0	0	0	0	0
HPV57	0	0	0	0	0	0	0	0	0	0	0	0	0	0	0	2	0
HPV59	0	0	0	0	0	0	0	0	0	1	0	0	0	0	0	0	0
HPV104	0	0	0	0	0	0	0	0	7	32	28	0	0	0	0	0	0
HPV105	0	0	0	0	0	0	0	0	0	1	0	0	0	0	0	0	0
HPV107	0	0	0	0	0	0	0	0	0	0	1	0	0	0	0	0	0
HPV109	0	0	0	0	0	0	0	0	0	0	0	3	0	0	0	0	0
HPV124	0	0	0	0	0	0	0	0	0	5	0	0	0	0	0	0	0
HPV138	0	0	0	1	0	0	0	0	0	0	0	0	0	0	0	0	0
HPV147	0	0	0	0	0	0	0	0	0	0	0	0	0	0	0	1	0
HPV155	0	0	0	0	0	0	0	0	156	1199	255	0	0	0	0	0	0
FA73	0	0	0	0	0	0	0	0	0	0	1	0	0	0	0	0	0
FA101	0	0	0	247	0	0	0	0	0	0	0	0	0	0	0	0	0
HPV 915 F 06 007 FD1	0	0	0	0	0	0	0	0	1	3	0	0	0	0	0	0	0
SE46	0	0	0	0	2	0	0	0	22	132	44	0	0	0	0	0	0
SE47	0	0	0	2	0	0	0	0	0	0	0	0	0	0	0	0	0
**Total HPV reads**	**0**	**0**	**0**	**250**	**2**	**0**	**0**	**0**	**378**	**2744**	**762**	**3**	**0**	**0**	**0**	**27**	**2**
Torque teno virus	0	0	0	0	0	1	12	51	1	1	1	0	0	0	1	0	0
Epstein barr virus	0	0	0	0	2	3	0	0	0	0	0	0	0	0	0	0	0
Human herpesvirus 8	0	0	0	1	0	0	0	0	0	0	0	0	0	0	0	0	0
Human polyomavirus 6	0	0	0	0	0	0	0	0	0	0	1	0	0	0	0	1	4
Merkel cell polyomavirus	0	0	0	0	0	0	22	0	1	2	1	0	0	0	0	0	0
HERV	1	1	0	1	1	0	0	0	0	3	0	1	0	0	0	1	1
**Total Virus reads**	**1**	**1**	**0**	**252**	**5**	**4**	**34**	**51**	**380**	**2750**	**765**	**4**	**0**	**0**	**1**	**29**	**7**

1 SCC = squamous cell carcinoma, AK = actinic keratosis, KA = keratoachantoma, WGA =  multiple displacement amplification ^2^Some samples were available in low volume and not included in all experiments.

We also identified sequences from two HPV types only distantly related to any known HPV types (designated as SE46 and SE47) (Table 2). Twenty-two reads from the pool of swab samples from the top of SCC and AK lesions formed the SE46 contig (GenBank accession number: JX198657), which was most closely related to the Gammapapillomavirus-type HPV112 in the E2 ORF of the genome. Two SE46 related singleton sequences were also found in fresh frozen samples from patients with KA. Ion Torrent 300 bp kit sequencing identified 132 reads related to SE46, which was assembled into two 950 bp and 3774 bp long contigs. SE47 was most closely related to the Alphapapillomavirus-type HPV94. Two SE47 singletons from the E6 and L2 parts of the genome were found in the pool of 72 paraffin embedded samples from patients with KA (GenBank accession numbers: JX198658, JX198659).

Checking of sequences for possible artifactual sequences composed of sequences from several different viruses (“chimeric sequences”), by comparing segments of the sequences for differential similiarities against the most closely related sequences in GenBank did not reveal any possible “chimeric” sequences.

### Sensitivity analysis

In order to obtain an approximate estimation of the sensitivity of the 454 HTS method, we analysed a clinical sample pool where eight reads of HPV16 were found. Real-time PCR determined that after WGA-amplification the sample contained 17 million copies of HPV16/microliter. We also performed real-time PCRs for HPV155 and SE46 on 142 clinical samples (Table 3). Only 4/142 and 5/142 samples were positive for HPV155 and SE46, respectively. The amount of virus present in the unamplified pool of these samples would have been 46,5 copies/2.5 ul input volume and 1,1 copy/2.5 ul input volume, respectively. All 142 samples were WGA-amplified separately and pooled after amplification. The amounts of HPV155 and SE46 in the pool were 660 000 and 3,3 million copies/ul, which resulted in 156 and 22 reads in the 454 sequencing, respectively (Table 2).

**Table 3:** Detection and viral loads of HPV155 and SE46 in individual samples.

		Viral copies/ cell		Viral copies/2.5 µl sample	
Diagnosis	No. of HPV155 positive patients	Biopsy of lesion	Biopsy of healthy skin	Swab from top of lesion	Swab from healthy skin
Squamous cell carcinoma (n = 89)	2	-	2.7×10^−5^	26.5	160.5
		-	3.8×10^−4^	10251	7235.5
		-	-	-	0.5
Actinic keratosis (n = 77)	3	1.8×10^−4^	-	6555^a^	7
		-	-	-	23
Basal cell carcinoma (n = 119)	2	-	-	40	112
		4.8×10^−5^	-	-	2.5
Seborrheic keratosis (n = 48) & Keratoacanthoma (n = 8)	0	-	-	-	-
	No. of SE46 positive patients				
		-	3.5×10^−4^	11	16.5
Squamous cell carcinoma (n = 89)	3	-	-	17	30
		-	-	-	2.5
		-	-	-	11.5
Actinic keratosis (n = 76)	3	-	-	13.5	110
		-	-	4.5	23.5
Basal cell carcinoma (n = 121) & Seborrheic keratosis (n = 47)	0	-	-	-	-
Keratoacanthoma (n = 8)	1	-	-	23.5	62

a Index sample from which HPV155 was amplified and cloned.

To investigate which amount of HPV16 copies per cell that would result in comparable amounts of virus after WGA amplification, we performed a titration series using the cervical cell line CaSki (containing about 500 copies HPV16/cell) diluted in HPV-negative C-33A cells. An input of 10% Caski cells was found to result in a concentration of about 165 million copies of HPV16 per microliter after whole genome amplification. 1% CaSki yielded 4.2 million copies/ul, 0.1% CaSki yielded 1,2 million copies/ul and 0.01% CaSki yielded about 220,000 copies/ul, suggesting that the 1% CaSki dilution (about 5 copies/cell) would have been detectable by the 454 sequencing.

As there appears to be a substantial variability in sensitivity, both in the WGA amplification and in the 454 sequencing step, the number of reads obtained by HTS should not be considered as a quantitation of the amount of virus present in the sample.

### Characterization of HPV155

**Figure 1:**
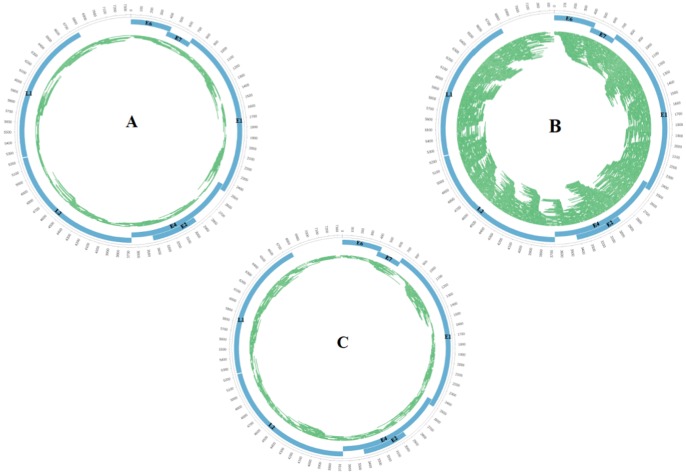
Coverage plots of HPV155 Genome from sequencing runs of (A) GSFLX 454, (B) Ion Torren PGM 300 bp kit and (C) Ion Torren PGM 400 bp kit.

The complete genome of HPV155 (SE42) was cloned from a swab sample from the top of an AK lesion. The clone and the sequence (obtained by primer walking) were sent to the International HPV Reference Center at the German Cancer Research Center in Heidelberg, where it was established as a new HPV type and given the designation HPV155 (GenBank accession JF906559). The HPV155 comprised 7352 bp and belonged to the genus Gammapapillomavirus but demonstrated only 76% similarity to the most closely related type (HPV149) (Table 4). In addition, sequencing of the HPV155-clone using the 454 FLX GS Junior and assembling with the Phred quality score cutoff of 30 (1 error in 1000 bases [41]) resulted in a 100% sequence identity to the sequence obtained by primer walking. To compare with the sequence quality from the original sample, we also re-assigned the 156, 1199 and 255 reads from the 454, Ion Torrent 300 bp and 400 bp kit sequencing runs, respectively related to HPV155 genome, to the sequence obtained by primer walking (Figure 1). Although these had been assembled with a Phred quality score of 15, still 145/156 reads, from 454, 1183/1199 from 300 bp Ion Torrent and 252/255 from 400 bp had high quality alignments (Figure 1). Two HPV155-related contigs from 454 and one contig from each Ion Torrent sequencing run showed 99,3% to 99,89% identities to the sequence obtained by primer walking.

**Table 4:** HPV155 genes and their similarity to the most closely related HPV type (HPV 149), as well as to HPV types representative of different species.

Gene(s)	Genome length	E6	E7	E1	E2	E4	L2	L1
Number of nucleotides (nt)*	7352	438	285	1821	1218	495	1557	1545
Position of ORF in genome	-	1–438	412–697	687–2507	2449–3666	2939–3433	3668–5224	5232–6779
Number of amino acids (aa)	–	145	94	606	405	164	518	514
	Alpha HPV16	44(23)	47(24)	57(12)	49(30)	44(16)	48(29)	58(49)
	Beta HPV38	48(28)	50(40)	60(47)	52(38)	46(22)	52(38)	61(55)
	Gamma-1 HPV4	54(36)	53(40)	63(53)	56(42)	54(27)	56(49)	63(59)
	Gamma-2 HPV50	54(38)	55(42)	61(46)	56(42)	52(30)	51(38)	61(57)
	Gamma-3 HPV48	51(37)	55(47)	59(47)	57(41)	52(23)	52(41)	61(57)
Sequence similarity percentage nt (percentage aa)	Gamma-4 HPV60	48(36)	54(34)	62(51)	53(42)	50(20)	56(44)	61(55)
	Gamma-5 HPV88	48(36)	55(42)	61(50)	56(42)	49(27)	55(45)	62(55)
	Gamma-6 HPV101	-	51(43)	61(47)	56(44)	54(27)	56(49)	63(62)
	Gamma-7 HPV109	65(55)	70(65)	76(72)	72(65)	71(57)	75(74)	74(80)
	Gamma-8 HPV112	56(37)	52(31)	60(45)	55(41)	52(31)	51(38)	63(59)
	Gamma-9 HPV116	50(35)	57(46)	61(46)	54(39)	50(20)	52(38)	60(54)
	Gamma-10 HPV121	52(36)	59(48)	64(53)	58(44)	53(27)	59(51)	66(61)
	Gamma unclassified HPV149	75(65)	79(76)	81(80)	76(70)	73(61)	76(77)	76(79)

* including STOP codon.

### Screening of HPV155 and SE46

Overall, HPV155 was detected among 7 of the 341 patients (Table 3). HPV155 was found in 0.6% (2/341) of both the lesions and the healthy skin biopsies. HPV155 was found in 1.1% (4/341) and 2.1% (7/341) of swab samples from top of lesion and healthy skin, respectively. Among the biopsies, the viral load of HPV155 was below 3.8×10^−4^ copies per cell. The quantity of HPV155 in the top of lesion swab samples varied between 40 to 10251 copies/2.5 ul whereas the corresponding figures from healthy skin were 0.5 to 7235 copies/2.5 ul (Table 3).

SE46 was detected in 7 of the 341 patients (Table 3). SE46 was found in one biopsy (0.2%, 1/341) from the healthy skin with a viral load of 3.5×10^−4^ copies per cell. SE46 was found in 1.5% (5/341) and 2.1% (7/341) of swab samples from top of lesion and healthy skin, respectively. The quantity of SE46 in the top of lesion swab samples varied between 11 to 23 copies/2.5 ul whereas the corresponding figures from healthy skin were 2.5 to 110 copies/2.5 ul (Table 3).

One SCC patient was positive for both HPV155 and SE46 in the biopsy of the healthy skin and in the swab samples from both tumour and normal skin, but the SCC biopsy was negative for both viruses. Eight patients with HPV155 or SE46 positive swab from top of the lesion were also positive in the corresponding swabs of healthy skin (Table 3).

## Discussion

The present study investigated the presence of HPVs in a variety of skin lesions using HTS, a method that is independent of any prior knowledge of virus sequences. The majority of the viral sequences found in this study (97%) originated from different HPVs. Most of them belonged to 19 previously known types or putative types, but two previously not described novel putative HPV types, SE46 and SE47, were also found.

Previously, we used 454 sequencing only after amplification of HPV sequences by broad general primer PCR [11]. The combined approach of broad general primer PCR and 454 sequencing of amplimers was highly efficient for detection of a multitude of viruses (subgenomic sequences from 110 previously known HPV types and from 44 novel putative HPV types were found) [11]. However, when the same samples were analysed in the present study two novel putative HPV types (SE46, SE47) were detected that had escaped detection when PCR was used prior to sequencing.

Although type-specific PCR with primers directed towards known sequences is unquestionably highly sensitive, it is debatable whether sensitive detection of very low amounts of virus is detecting a relevant infection. Cleansing of the skin surface with tape will remove most of the HPV positivity without detectably altering the skin architecture [21], suggesting that many HPV DNA positivities may reflect skin surface contamination that has not established an infection. Indeed, transcriptome sequencing of a series of HPV DNA-positive skin cancers did not find viral RNA expression [50], suggesting that the viral DNA detected did not reflect an infection in the lesion.

Foulongne and collegues used a similar unbiased approach as our investigation and reported a large number of sequence reads, including nine novel HPVs, when sequencing six samples from healthy forehead skin [12]. The larger amount of viral reads found compared to our study was most likely due to the fact that these authors used the Illumina HiSeq-2000 sequencing platform that can produce considerably more reads than the 454 system [51]. However, we opted for the 454 system, because the longer read length (∼450 bp in 454 system and ∼100 bp in Illumina) greatly facilitates a reliable bioinformatics assembly of raw reads to contigs, in particular for de novo assembly of new viruses where a reference sequence is not available. Foulongne and collegues used only forehead swabs, known to be a rich source of HPV DNA [52]. We used both swab samples, fresh-frozen biopsies from stripped skin and formalin-fixed paraffin-embedded lesions and report that viral sequences could be obtained from all these types of skin sample [21]. Comparison with the Ion Torrent systems found that their 300 bp version produced seven times more reads than 454 and that their 400 bp version produced about twice as many reads as 454 did. Thus, there is a trade-off between sequencing depth and sequencing length and the optimal choice may depend on whether the bioinformatics step can accurately handle short reads without risk for mis-assembly.

The skin cancer pool with samples that had previously been HPV positive by PCR [29], but that was now HPV-negative in the sequencing, had largely contained very low viral loads probably below the limit of detectability of the sequencing approach. The high cost of HTS and a tendency for variability in WGA efficiency precluded a more exact definition of the limit of detectability of the 454 sequencing approach, although it appears that samples with >5 copies of virus/cell would definitely be detectable. Increasing the sequencing depth using the Ion Torrent did detect seven additional known or putative HPV types, with low amount of reads, although no additional new HPV types were identified. It is notable that our sequencing detected SE46 in a pool of 142 samples where our type specific real-time PCR found that only five patients in the pool were positive for SE46 and only in moderate copy numbers. Also, the HTS detected HPV109 in a pool of skin cancer samples that had been negative for HPV by PCR [29]. HPV109 has several primer mismatches to the HPV general primer system used and is thus amplified poorly by this PCR, further demonstrating the advantage of our PCR-free method as this will detect the most abundant viruses present without being biased by the PCR primer sequences used.

Our methods that separated viral DNA from human DNA before WGA and sequencing were less successful for detecting viral reads than directly subjecting samples to WGA and sequencing. Even though the fractions collected from the ultracentrifugation had been carefully evaluated to contain viral particles and no human DNA (using control experiments with samples spiked with HPV 16 pseudovirions and realtime PCRs for viral and human DNA [29]) only 51 viral reads were detected. Conceivably, handling of low amounts of viral DNA may result in loss of material.

As specimens may contain several closely related HPV types, the possibility exists that assembly algorithms may construct erroneous “chimeric” sequences by the assembly of 2 different sequences from different viruses. To prevent incorrect assemblies, we processed only reads having at least 80% of base pairs with quality score of ≥15 (base calling accuracy of 97%). Furthermore, we used stringent assembly parameters in the CABOG assembler and also manually investigated all viral sequences for possible miss-assemblies by assessing consensus quality scores. Also we used python (www.python.org) scripts to check for possible “chimeric sequences” by comparing segments of the sequences for differential similarities against the most closely related genomes in GenBank.

Screening for the new HPV155 and putatively new SE46 among patients with SCC, AK, BCC, SK and KA revealed only a few positive patients. There were low viral loads in biopsies, but the swab samples of some patients contained high copy numbers (up to 10251 copies per 2.5 ul). Presence of virus on top of lesions, but not in the lesions themselves, is suggestive of virus production at a site different from where the biopsy was taken.

The two putative novel HPV types reported in this study (SE46 and SE47) belonged to the Gammapapillomavirus and Alphapapillomavirus genera, respectively. Gammapapillomavirus is a rapidly growing HPV genus with at least 58 completely characterized types/genomes (including HPV155) [6], [8]–[10], [12] and 119 subgenomic sequences detected using the general primer PCR FAP (FA-types), augmented with HTS (SE types) [11]. Detection of new types within the genus Alphapapillomavirus is nowadays less common. Most Alphapapillomavirus types are mucosal, but Alphapapillomavirus species 2, 4 and 8 are also found in cutaneous lesions. HPV94, which is the known virus most closely related to SE47, belongs to species Alphapapillomavirus-2.

The previously described putative type FA101 had a partial 431 bp sequence deposited in GenBank. As it was abundant in the pooled formalin-fixed paraffin embedded KA samples, we now report a complete viral genome of 7359 bp of the FA101 sequence. Another useful result of the sequencing was the identification of the complete HPV155 sequence. This was used to design primers for cloning and subsequent Sanger sequencing which confirmed the HTS-obtained sequence of this virus.

## Conclusions

High throughput sequencing of skin lesions was useful for an unbiased assessment of viral DNA in these lesions. Most of the viral DNA was found to represent known HPV types or known subgenomic sequences of HPV.
